# P26 Exploring socioeconomic disparities effect on patient understanding of antimicrobial resistance: a Southwest Devon study

**DOI:** 10.1093/jacamr/dlae136.030

**Published:** 2024-08-23

**Authors:** Kerim Meftahpour, Rishika Segireddy, Alexandra Macrostie, Evangeline Hartley, Lewis Bond-Kendall, Marcus Walch, Raveena Marwaha

**Affiliations:** University of Plymouth, Plymouth, UK; University of Plymouth, Plymouth, UK; University of Plymouth, Plymouth, UK; University of Plymouth, Plymouth, UK; University of Plymouth, Plymouth, UK; University of Plymouth, Plymouth, UK; University of Plymouth, Plymouth, UK

## Abstract

**Background:**

With projections indicating 10 million annual deaths worldwide by 2050 due to AMR,^1^ understanding public perceptions is crucial. Primary care settings, where antibiotic prescribing is prevalent (>81% in the UK),^2^ are especially important. Assessing the impact of deprivation on patient behaviours is essential. St Levan Surgery (PL21JR) and Buckfastleigh Medical Centre (TQ110DE) represent areas with varying levels of deprivation. St Levan Surgery (PL2 1JR) is surrounded by an area of 1st to 4th decile for Index of Multiple Deprivation, contrasting with Buckfastleigh Medical Centre’s (TQ110DE) 4th to 7th decile.^3^ Investigating differences between these primary care services can provide insight into the socioeconomic disparities affecting AMR awareness and stewardship.

**Objectives:**

This cross-sectional study investigates patient perceptions of antimicrobial resistance (AMR) across two differing socioeconomic areas in Southwest Devon. The aim is to assess patient knowledge of AMR, attitudes towards antibiotic use and factors influencing behaviours to better understand the effect of deprivation on AMR awareness and understanding and potentially inform targeted interventions for mitigating AMR in primary care.

**Methods:**

A questionnaire was administered to patients prescribed antibiotics in the past six months through face-to-face interactions, QR codes and NHS-approved texting services. Responses were collected from 72 patients at St Levan Surgery (November 2023) and 110 patients at Buckfastleigh Medical Centre (March 2024).

**Results:**

Analysis revealed disparities in patient awareness and antibiotic usage between the two regions. *Awareness of treatable infections:* Patients at St Levan showed lower awareness, especially regarding fungal infections such as thrush. *Awareness of AMR*: Buckfastleigh patients exhibited higher awareness, indicating potential knowledge gaps in St Levan. *Understanding of AMR causes*: Neither group showed substantial awareness, highlighting the need for educational interventions. *Antibiotic prescribing discrepancies*: Buckfastleigh patients received more antibiotic prescriptions, possibly influenced by patient characteristics and healthcare provider behaviours.

**Conclusions:**

While Buckfastleigh exhibited higher overall awareness, both areas could benefit from addressing gaps in understanding AMR causes and treatable infections. The study emphasizes the necessity for tailored interventions, particularly in deprived areas such as St Levan Surgery, to enhance patient knowledge and combat AMR effectively. Differences in prescribing practices should also be further investigated and may be related to the inverse care law.
